# *Astragalus* and *Paeoniae* Radix Rubra extract (APE) inhibits hepatic stellate cell activation by modulating transforming growth factor-β/smad pathway

**DOI:** 10.3892/mmr.2014.3026

**Published:** 2014-12-01

**Authors:** WEIJUAN HUANG, LIN LI, XIAOPENG TIAN, JINJIN YAN, XINZHENG YANG, XINLONG WANG, GUOZHEN LIAO, GENQUAN QIU

**Affiliations:** 1 Department of Scientific Research, Xi’an Medical College, Xi’an, Shaanxi 710061, P.R. China; 2 State Key Laboratory of Oncology in South China, Collaborative Innovation Center of Cancer Medicine, Sun Yat-sen University, Guangzhou, Guangdong 510060, P.R. China; 3 Department of Pharmacology, Xi’an Medical College, Xi’an, Shaanxi 710061, P.R. China; 4 Department of Traditional Chinese Medicine, First Affiliated Hospital of Xi’an Jiao Tong University, Xi’an, Shaanxi 710061, P.R. China

**Keywords:** *Astragalus* and *Paeoniae* Radix Rubra extract, transforming growth factor-β/smad pathway, plasminogen activator inhibitor type 1, urokinase-type plasminogen activator

## Abstract

Previous studies have shown that *Astragalus* and *Paeoniae* Radix Rubra extract (APE) is capable of protecting against liver fibrosis in rats. The hypothesis of the present study was that APE exerts its anti-fibrotic effect by mediating the transforming growth factor β (TGF-β)/Smad signaling pathway. In order to investigate this hypothesis, a series of assays were designed to detect the effects of APE on cell proliferation, cell invasion and the activation of hepatic stellate cells (HSCs). In addition, the effects of APE on the TGF-β/Smad signaling pathway were explored, with the aim of elucidating the underlying mechanisms. HSCs were initially isolated from normal rat liver. A number of assays were then employed in order to evaluate the effects of APE on the function of these cells. Cell proliferation was investigated using an MTT assay and cell invasion was observed with the use of transwell invasion chambers. Collagen synthesis was measured with a ^3^H-proline incorporation assay and expression of α-smooth muscle actin was used to determine the extent of HSC activation. Protein expression induced by TGF-β1 in HSCs was investigated by western blot and immunofluorescence analyses. Plasminogen activator inhibitor type1 (PAI-1) and urokinase-type plasminogen activator (uPA) transcriptional activity was measured using reverse transcription polymerase chain reaction. The results demonstrated that APE (5–80 μg/ml) significantly inhibited fetal bovine serum-induced cell proliferation in a dose-dependent manner. Cell invasion and activation of HSCs induced by TGF-β1 were disrupted by treatment with APE in a dose-dependent manner. TGF-β1 was observed to increase the phosphorylation of Smad2/3, while APE administered at higher doses produced inhibitory effects on Smad2/3 phosphorylation. In addition, administration of APE abrogated the TGF-β1-induced reduction in Smad-7 expression in a dose-dependent manner. The results further indicated that APE treatment not only reduced PAI-1 expression, but also increased uPA expression in a dose-dependent manner. In conclusion, APE exerted inhibitory effects on cell proliferation, invasion and activation of HSCs, and the mechanisms underlying these effects may involve the TGF-β1/Smad pathway.

## Introduction

Hepatic fibrosis (HF) is recognized as one of the most common types of liver disease, as well as one that is resistant to the majority of current therapies, resulting in significant global morbidity (1). HF has been defined as a tissue-specific response to long-term injury or illnesses, including chronic viral hepatitis, alcoholic liver disease, cholestasis, circulatory disturbances, autoimmune liver disease or one of a number of nutritional disorders (2,3). Liver fibrosis is characterized by the excessive deposition of extracellular matrix (ECM) proteins, consisting predominantly of type I and type III collagen. These abnormal depositions disturb the structure of the hepatic lobule, misdirecting blood flow in the liver and thereby disturbing its healthy functioning. This leads to liver cirrhosis and, ultimately, to liver carcinoma (4). Although numerous therapeutic options are currently available for liver fibrosis, all have limited degrees of success and none were capable of producing a complete cure (5). Thus, there is an urgent need to develop better preventative options as well as treatment approaches, based on a more thorough understanding of the pathogenesis of hepatic fibrosis.

Although the exact pathophysiological mechanisms underlying the formation of hepatic fibrosis are elusive, there are a number of potential processes that may be worthy of investigation. Hepatic stellate cells (HSCs) are an important type of fibrogenic liver cell. They are found during liver injury and are known to be responsible for the progression of hepatic fibrosis (6). These cells may be activated, which induces their transdifferentiation into myofibroblasts (MFBs). MFBs are characterized by a number of fibrotic functions, including the induction of ECM deposition, α-smooth muscle actin (α-SMA) expression, as well as the synthesis and secretion of type I and type III collagen (7,8). A growing body of evidence has documented that inhibition of the transformation of HSCs may aid in the prevention and cure of liver fibrosis (9). However, HSCs are not the only mechanism through which fibrosis progresses. A number of studies have indicated that this process is complicated and involves numerous cytokines and signaling pathways (10,11).

Transforming growth factor (TGF)-β1 has been identified as the most significant factor involved in the activation and promotion of the transformation of HSCs (12). Previous studies have demonstrated that TGF-β1 is highly expressed in numerous tissues, which exhibit fibrosis. Furthermore, TGF-β1 has been shown to have a key role in the development of fibrosis by promoting the proliferation of tissue fibroblasts and the synthesis of collagen (13). The following stages of the cell-signaling pathway of TGF-β1 have been elucidated: The initial step is the transmission of a signal from the activated transmembrane receptor, TβR. This signal is then transmitted to the nucleus, predominantly through intracellular members of the Smad family. Further evidence for this mechanism has been provided by studies in which the TGF-β/Smad pathway was inhibited via RNA interference. This resulted in a significant decrease of type I and III collagen expression in fibrotic livers (14). Based on these findings, subsequent studies have confirmed the potential use of members of the Smad family as biomarkers with which to predict liver disease progression (15). Thus, the TGF-β/Smad pathway is hypothesized to be a target for the treatment and cure of hepatic fibrosis.

*Astragalus* and *Paeoniae Radix Rubra extract* (APE) is produced from a variety of herbs (*Astragali* Radix, *Paeoniae* Radix Rubra, *Curcumae* Rhizoma, Bupleuri Radix and *Eupolyphaga*) with a standard ratio of 30:30:15:12:10 by crude herb weight and contains the active components paeoniflorin, astragalosides and curzenone (16). *Astragali* Radix is thought to have restorative properties in Chinese Medicine, and studies have demonstrated that it is a hepatoprotective agent (17,18). Extracts from *Paeoniae* Radix Rubra, *Curcumae* Rhizoma, Bupleuri Radix and *Eupolyphaga* also have the potential to suppress liver fibrosis and have been considered potent herbs with which to treat liver disease (19). Previous *in vivo* experiments have been conducted, which demonstrated that APE administered at a ratio of 30:30:15:12:10 exhibited superior antifibrotic effects compared to administration of individual herbs in a model of CCl_4_-induced chronic liver injury (16). However, the molecular mechanisms underlying the hepatoprotective effects of APE have remained elusive. Therefore, the present study aimed to investigate the effects of APE on proliferation, invasion and activation of HSCs. In addition, the mechanisms underlying the effect of APE on the TGF-β/Smad pathway were examined.

## Materials and methods

### Preparation of APE

The following herbs were purchased at Xi’an Chinese Medicine Corporation (Xi’an, China): *Astragali* Radix, *Paeoniae* Radix Rubra, *Curcumae* Rhizoma, *Bupleuri* Radix and *Eupolyphaga*. Each herb was identified by Dr Genquan Qiu, an author of the study and a specialist in Traditional Chinese Herbal Medicine. Herb samples were preserved in the specimen room of the Institute of Clinical Pharmacology at the Xi’an Medical College (Xi’an, China). The process of extracting and preparing the APE components from the five herbs was as follows: A total amount 8.45 kg dried sliced crude herbs (*Astragali* Radix*, Paeoniae* Radix Rubra, *Curcumae R*hizoma*,* Bupleuri Radix and *Eupolyphaga*) in a standard ratio of 30:30:15:12:10 were decocted in 80 l water three times at 95°C for 35 min. The decocted solution was filtered though 150 μm gauze ( Sigma-Aldrich, St. Louis, MO, USA) and the combined filtrates were then concentrated to a mass of 4.22 kg in a vacuum desiccator at 70°C. The sediment was dried into power using a spray drier at a temperature range of 80–160°C. This process yielded 2.11 kg of dry powder. It should be noted that in all subsequent *in vitro* cell experiments, APE powder was dissolved in Hank’s solution (Sigma-Aldrich).

### Cell culture

Male Sprague-Dawley rats were obtained from the Experimental Animal Center of Anhui Medical University (Anhui, China) and had a weight range of 160–200g. HSCs were separated from normal rat liver and maintained in Dulbecco’s modified Eagle’s medium (DMEM; Sigma-Aldrich), supplemented with 10% fetal bovine serum (FBS; Sigma-Aldrich) (20). Cells were cultured in an environment of 95% air and 5% CO_2_ at 37°C. All experiments were performed during the exponential growth phase, once the cells had been plated for 24 h.

### Cell proliferation assay

HSCs were seeded in 96-well plates at a density of 1×10^4^ cells/well and cultured in 200 μl DMEM supplemented with 10% FBS. Following overnight starvation in serum-free medium, HSCs were incubated with 10% FBS with various concentrations of APE (5, 10, 20, 40 or 80 μg/ml) for 16 h. Control conditions consisted of cells which had an equal volume of serum-free medium added without the addition of APE. Following incubation, cell proliferation was assessed using an MTT assay (Abcam, Cambridge, UK), as previously described (21). Absorbance was measured in an ELISA microplate reader at 562 nm (FlexStation 3; Molecular Devices, Sunnyvale, CA, USA). Data are expressed as the mean of a minimum of three independent experiments.

### Cell invasion assay

Transwell invasion chambers with 8-μm membrane pores coated with Matrigel™ (BD Biosciences, San Jose, CA, USA) were placed in 24-well plates. HSCs were starved overnight in serum-free DMEM. Cells were then seeded in the upper compartment of the chamber at a concentration of 1×10^5^ cells/well and incubated with or without the administration of APE at various concentrations (10, 20, 40 or 80 μg/ml) for 24 h. Medium containing TGF-β1 (40 pmol/l) was placed into the lower compartment of the chamber. Serum-free medium was used for the control group. Following incubation, cells on the upper side of the membrane were completely removed using a cotton swab. Cells which had crossed the Matrigel barrier and migrated to the lower side of the chamber were fixed with 100% methanol for 1 min and stained with hematoxylin and eosin (H&E; Sigma-Aldrich). The number of invading cells was counted in five randomly selected fields at ×100 magnification (CX31; Olympus Corp., Tokyo, Japan). The mean number of invading cells in these fields was calculated and the experiments were run in triplicate.

### Collagen synthesis assay

Collagen synthesis was evaluated using a ^3^H-proline incorporation assay (22). In brief, HSC cells were seeded in 96-well plates at a density of 1×10^4^ cells per well for at least 24 h. Cells were synchronized by culturing in serum-free medium overnight prior to incubation with 40 pmol/l TGF-β1 in the presence of APE at various concentrations (10, 20, 40 or 80 μg/ml) for 24 h. The control group received an equal volume of serum-free medium without APE. The aforementioned cultures were subsequently exposed to ^3^H-proline (37 kBq; Atom-Hitech, Beijing, China) for 12 h. Following proline exposure, the cells were washed twice with phosphate-buffered saline (PBS; Sigma-Aldrich), treated with ice-cold 5% trichloroacetic acid (Sigma-Aldrich) for 1 h and then washed twice more with distilled water. Finally, cells were lysed with 0.25% trypsogen and counted in a liquid scintillation counter (LS6000SE; Beckman Coulter, Fullerton, CA, USA).

### Immunoblot analysis

HSCs were seeded at a density of 1×10^6^ cells per dish. Once they reached sub-confluency, the cells were cultured in serum-free medium for 24 h in the presence or absence of APE at various concentrations (10, 20 or 40 μg/ml). Cells were then treated with 40 pmol/l TGF-β1 for 30 min. Control cells were treated with neither TGF-β1 nor APE. Following incubation, cells were homogenized using a modified radioimmunoprecipitation assay buffer (50 mM Tris-HCl, pH 7.4; 1% NP-40; 150 mM NaCl; and 1 mM EDTA; Sigma-Aldrich) supplemented with protease and phosphatase inhibitors (1 mM phenylmethyl sulfonyl fluoride, 0.1 mM *N*-tosyl-L-phenylalanine chloromethyl ketone, 1 mg/ml aprotinin, 1 mg/ml pepstatin, 0.5 mg/ml leupeptin, 1 mM NaF, 1 mM Na_4_P_2_O_4_ and 2 mM Na_3_VO_4;_ Sigma-Aldrich). The extract was centrifuged at 16,000 × g for 30 min at 4°C in order to remove cell debris. The supernatant was collected and quantified using the bicinchoninic acid protein assay (Pierce Biotechnology, Rockford, IL, USA) and boiled for 5 min with SDS sample buffer (100 mM Tris-HCl, pH 6.8; 4% SDS; 12% β-mercaptoethanol; 20% glycerol; and 0.01% bromophenol blue; Sigma-Aldrich) at the equivalent protein level. Samples were subjected to SDS-PAGE and transferred to polyvinylidene difluoride membranes (Bio-Rad Laboratories, Hercules, CA, USA). The membranes were then blocked with 10% skimmed milk powder (NanRong International Corp., Kaohsiung, Taiwan) in PBS containing 0.1% Tween-20 (Sigma-Aldrich) and incubated with the following primary antibodies: Mouse anti-human monoclonal antibodies against PAI-1 (1:2,000 mouse monoclonal, ab125687) and uPA (1:1,500, mouse monoclonal, ab82220), which were purchased from Abcam; mouse anti-human monoclonal antibodies against TGF-β1 (1:1,000, mouse monoclonal, sc-130348) Santa Cruz Biotechnology, Inc., Dallas, TX, USA); rabbit anti-human phospho-Smad2 antibody (1:500, mouse monoclonal, BS3725), rabbit anti-human Smad2 antibody (1:500, mouse monoclonal, BS2993), rabbit anti-human phospho-Smad3 antibody (1:500, mouse monoclonal, BS4874), rabbit anti-human Smad3 antibody, (1:500, polyclonal, AP0446), rabbit anti-Smad7 antibody (1:500, polyclonal, BS60366) and rabbit anti-β-actin antibody (1:4,000, polyclonal, AP0060), which were purchased from Bioworld Technology (St. Louis, MO, USA); as well as rabbit anti-α-SMA antibody (1:2,000, polyclonal, BS70000; Cell Signaling Technology, Danvers, MA, USA), overnight at 4°C. Following application of the primary antibodies, membranes were incubated with the appropriate secondary antibodies against mouse and rabbit (mouse IgG, 1:2,000, sc-2025; rabbit IgG 1:2,000, sc-2027; Santa Cruz Biotechnology, Inc.) for 2 h at room temperature. Finally, immunoreactivity was visualized using enhanced chemiluminescnce (Amersham Pharmacia Biotech, Picastaway, NJ, USA) and autoradiography. The resulting images were subjected to densitometric analysis using Quantity One software (Bio-Rad Laboratories).

### Immunofluorescence

HSCs were seeded onto 24-well plates at 5×10^3^ cells per well. Cells were then incubated in serum-free medium for 24 h with or without treatment with APE at various concentrations (10, 20 or 40 μg/ml) and stimulated with 60 pmol/l TGF-β1 for 1 h. Control cells were incubated in serum-free medium with neither TGF-β1 nor APE. Following incubation, cells were fixed with 4% paraformaldehyde for 30 min, permeabilized and then blocked with 0.1% saponin and 0.5% bovine serum albumin (Sigma-Aldrich) in PBS for 30 min at 4°C. Cells were incubated overnight at 4°C with the primary antibodies described above, and then incubated with fluorescein isothiocyanate-conjugated goat anti-rabbit immunoglobulin G (1:100) for 2 h at room temperature. Slides were then mounted with 80% phosphoglycerol, and visualized under a fluorescence microscope (CX31; Olympus Corp.). In each experiment, five randomly selected fields were analyzed from each sample.

### Reverse transcription-polymerase chain reaction (RT-PCR)

HSCs were seeded at 1×10^6^ cells per dish. Cells were cultured in serum-free medium for 24 h with or without treatment with APE at various concentrations (10, 20 or 40 μg/ml) treatment and stimulated with 10 pmol/l TGF-β1 for 3 h prior to harvesting. Control cells were cultured in an equal volume of serum-free medium without TGF-β1 stimulation. The mRNA levels of HSCs were analyzed using an RT-PCR assay. Total sample mRNA was extracted using Trizol reagent (Invitrogen Life Technologies, Carlsbad, CA, USA) according to the manufacturer’s instructions. Revert Aid First Strand cDNA Synthesis kit (Fermentas, Vilnius, Lithuania) was used to produce corresponding cDNA. The primers for plasminogen activator inhibitor type 1 (PAI-1), urokinase-type plasminogen activator (uPA) and β-actin (Sangon Biotech, Shanghai, China) were as follows: Forward, 5′-CGGAGCACGGTCAAGCAAGTG-3′ and reverse, 5′-GTTGAGGGCAGAGAGAGGCGC-3′ for PAI-1; forward, 5′-ACTACTACGGCTCTGAAGTCACCA-3′ and reverse, 5′-GAAGTGTGAGACTCTCGTGTAGAC-3′ for uPA; and forward, 5′-CTCCATCCTGGCCTCGCTGT-3′ and reverse, 5′-GCTGTCACCTTCACCGTTCC-3′ for β-actin. All target sequences were separately amplified for 30–35 cycles of 30 sec at 94°C, 30 sec at 55°C and 60 sec at 72°C. Samples of each reaction product were separated by agarose gel electrophoresis (Sigma-Aldrich), visualized by ethidium bromide staining (Sigma-Aldrich) and visualized using 290 nm ultraviolet illumination (E1617-T130; Bio-Rad Laboratories). The density of each band was measured by densitometry using Quantity One 4.52 software (Bio-Rad Laboratories).

### Statistical analyses

Statistical analysis was performed using SPSS software (SPSS standard version 17.0; SPSS, Inc., Chicago, IL, USA). Data are expressed as the mean ± standard deviation. Comparisons between groups were made using Student’s t-test and the Mann-Whitney rank sum test, the latter of which was used to compare the degree of staining intensity. P<0.05 was considered to indicate a statistically significant difference.

## Results

### APE suppresses HSC proliferation induced by FBS

In order to detect the effect of APE on HSCs proliferation, cells were stimulated with 10% FBS and treated with varying concentrations of APE (5–80 μg/ml). As shown in Table I, 10% FBS increased the proliferation of HSCs, while the administration of APE (5–80 μg/ml) resulted in a suppression of HSC proliferation. The effect of APE-induced HSC suppression occurred in a dose-dependent manner, with an IC_50_ of 22.45 μg/ml.

### APE inhibits cell invasion induced by TGF-β1

**Figure 1:**
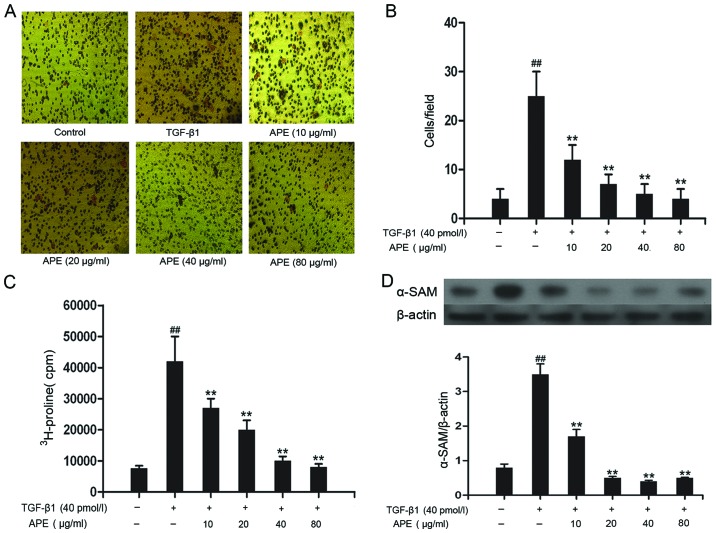
Effects of APE on TGF-β1-induced invasion and activation of HSCs. (A and B) Number of cells resulting from stimulation of HSCs by TGF-β1 treatment exhibited a significant, dose-dependent suppression when treated with APE (10, 20, 40 or 80 μg/ml) (magnification, ×100). (C) TGF-β1-stimulated HSC collagen synthesis was significantly and dose-dependently decreased following treatment with APE (10, 20, 40 or 80 μg/ml). **(**D**)** Expression of α-SAM was significantly decreased following treatment with APE (10, 20, 40 or 80 μg/ml) compared with that in the control. ^##^P<0.05 compared with control group and ^**^P<0.05 compared with group treated with TGF-β1 alone. APE, *Astragalus* and *Paeoniae* Radix Rubra extract; TGF-β1, transforming growth factor-β1; α-SAM, α-sterile alpha motif; HSC, hepatic stellate cells.

The invasion capability of HSCs was investigated using a Transwell invasion assay. As shown in Fig. 1A, HSCs moved to the lower compartment of the chambers across the Matrigel-coated polycarbonate membrane when stimulated by treatment with TGF-β1. Treatment with APE (10, 20, 40, 80 μg/ml) significantly reduced the number of TGF-β1-stimulated cells that invaded across the polycarbonate membrane. In accordance with previous results, this effect occurred in a dose-dependent manner (23). Thus, TGF-β1-induced HSC invasion into the bottom chamber and across the collagen IV/collagen I-coated polycarbonate membrane was inhibited by APE (Fig. 1A and B).

### APE reduces TGF-β1-induced HSC activation

Collagen synthesis was measured using a ^3^H-proline incorporation assay. As shown in Fig. 1C, collagen synthesis in TGF-β1-stimulated HSCs was markedly increased compared with that of control cells (P<0.01). Furthermore, treatment with APE (10, 20, 40, 80 μg/ml) significantly reduced TGF-β1-induced collagen synthesis in a dose-dependent manner. Protein expression of α-SAM was then used to measure HSC activation. As shown in Fig. 1D, the expression of the α-SAM protein following treatment with TGF-β1 alone was 3.5-fold higher than that in the control group. Furthermore, administration of APE (10, 20, 40, 80 μg/ml) decreased α-SAM expression in a dose-dependent manner. These results indicate that APE inhibits liver fibrosis by suppressing HSC activation and collagen synthesis.

### APE reduces TGF-β1-induced receptor regulated (R)-Smad phosphorylation in HSCs

**Figure 2:**
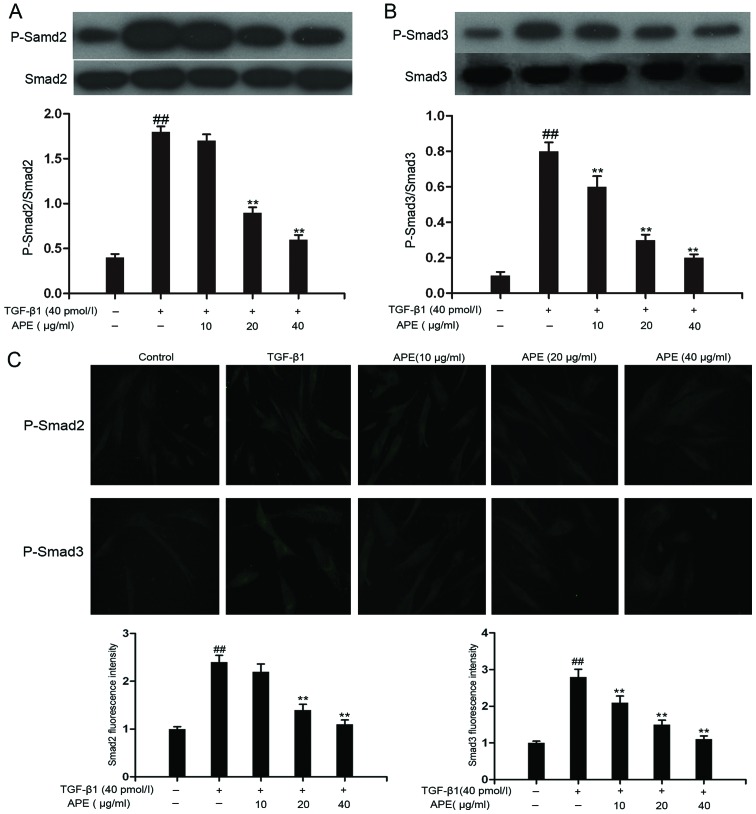
Effects of APE treatment on TGF-β1-dependent Smad2 and Smad3 phosphorylation in HSCs. (A) TGF-β1 significantly increased levels of Smad-2 phosphorylation, contrasting with the suppression of the Smad-2 phosphorylation following treatment with higher doses of APE (20 or 40 μg/ml).(B) TGF-β1 enhanced Smad-3 phosphorylation, and this increase was suppressed by treatment with APE (10, 20, 40 μg/ml) in a dose-dependent manner. (C) Expression of phosphorylated R-Smad was elevated following TGF-β1 treatment. Treatment with APE (20, 40 ug/ml) significantly decreased the fluorescence intensity of phosphorylated Smad2 and Smad3 in a dose-dependent manner. ^##^P<0.05 compared with control group and ^**^P<0.05 compared with the group treated with TGF-β1 alone. APE, *Astragalus* and *Paeoniae* Radix Rubra extract; TGF-β1, transforming growth factor-β1; HSC, hepatic stellate cells.

R-Smad phosphorylation was detected by western blot and immunofluorescence analyses. As shown in Fig. 2A and B, western blotting results demonstrated that TGF-β1 significantly increased the levels of Smad-2 phosphorylation and moderately enhanced Smad-3 phosphorylation. This increase in R-Smad phosphorylation was subsequently suppressed by the administration of APE (20, 40 μg/ml) in a dose-dependent manner. Of note, a 10 μg/ml dosage of APE significantly reduced Smad-3, but not Smad-2 phosphorylation. Immunofluorescence also demonstrated that the expression of phosphorylated R-Smad was elevated as a result of TGF-β1 treatment (Fig. 2C). Treatment with APE (20, 40 μg/ml) significantly reduced the fluorescence intensity of phosphorylated Smad2 and Smad3 in a dose-dependent manner. In accordance with the results from the western blotting experiments, a 10 μg/ml dosage of APE reduced the fluorescence intensity of phosphorylated Smad3, but had no detectable effect on that of phosphorylated Smad-2. These results indicated that APE decreased TGF-β1-induced R-Smad phosphorylation.

### APE enhances Smad7 expression in HSCs, reversing downregulation of Smad7 by TGF-β1

**Figure 3:**
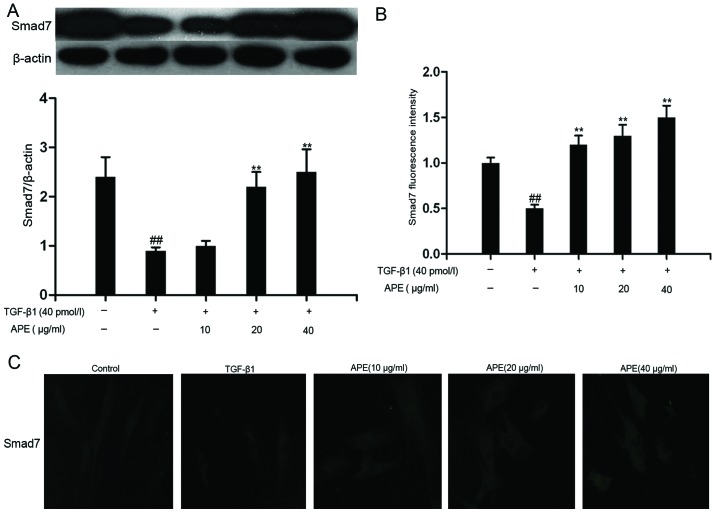
Effects of APE on TGF-β1-induced reduction in Smad7 expression in HSCs. (A) Protein expression of Smad7 was significantly reduced following treatment with TGF-β compared with that in controls. Higher doses of APE (20 or 40 μg/ml) were shown to increase Smad7 expression. (B and C) TGF-β1 decreased Smad7 fluorescence intensity, whereas higher doses of APE (20 or 40 μg/ml) increased Smad7 fluorescence intensity. ^##^P<0.05 compared with control and ^**^P<0.05 compared with group treated with TGF-β1 alone. APE, *Astragalus* and *Paeoniae* Radix Rubra extract; TGF-β1, transforming growth factor-β1; HSC, hepatic stellate cells.

As shown in Fig. 3A, western blot analysis demonstrated that the protein expression of Smad7 was significantly reduced following treatment with TGF-β1 compared with that in the controls. However, administration of high doses of APE (20 and 40 μg/ml) was able to inverse this effect, leading to an increase in the expression of Smad7. Immunofluorescence demonstrated that TGF-β1 reduced the fluorescence intensity of Smad7, whereas treatment with APE (10, 20 or 40 μg/ml) increased Smad7 fluorescence intensity (Fig. 3B and C). These results indicated that APE at high doses increased the expression of Smad-7.

### APE suppresses PAI-1 and increases uPA expression, reversing the effects of TGF-β1 on HSCs

**Figure 4:**
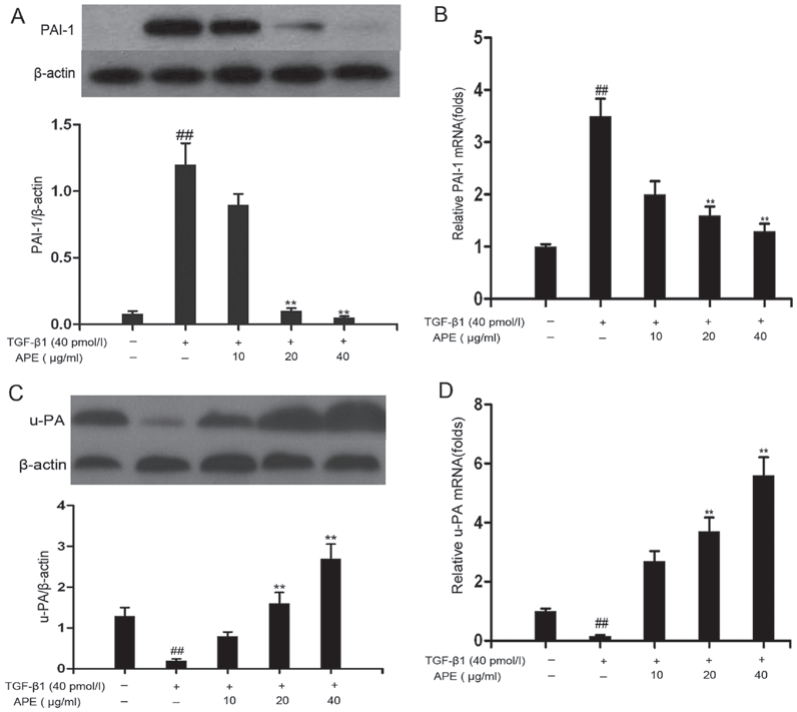
Effects of APE on PAI-1 and uPA in TGF-β1-induced HSCs. (A) Protein expression of PAI-1 was markedly decreased in HSCs compared with that in controls treated with TGF-β1 alone. APE treatment (20 or 40 μg/ml) was shown to suppress protein expression in a dose-dependent manner. (B) TGF-β1 treatment induced an increase of ~3.5-fold in PAI-1 mRNA expression compared with controls. Treatment with APE was found suppress the transcriptional activity of PAI-1 in a dose-dependent manner. (C) TGF-β1 strongly decreased uPA protein levels in HSCs. Treatment with APE completely restored uPA protein expression at concentrations of either 20 or 40 μg/ml. (D) The transcriptional activity of uPA was found to be lower following TGF-β1 treatment. Application of APE at dosages of 20 and 40 μg/ml were found to increase mRNA levels of uPA. ^##^P<0.05 compared with control and ^**^P<0.05 compared with group treated with TGF-β1 alone. APE, *Astragalus* and *Paeoniae Radix Rubra* extract; PAI-1, plasminogen activator inhibitor type 1; uPA, urokinase-type plasminogen activator; TGF-β1, transforming growth factor-β1; HSC, hepatic stellate cells.

As shown in Fig. 4A, protein expression of PAI-1 was markedly increased in HSCs treated with TGF-β1 compared with that in the control group. However, treatment with APE (20 or 40 μg/ml) suppressed protein expression in a dose-dependent manner. TGF-β1 induced an increase of ~3.5-fold in PAI-1 mRNA expression compared with controls, while APE suppressed the transcriptional activity of PAI-1 is a dose-dependent manner. This inhibitory effect of APE was statistically significant at higher concentrations (20 and 40 μg/ml; Fig. 4B). In accordance with these results, TGF-β1 significantly reduced uPA protein levels in HSCs, while APE, at concentrations of 20 or 40 μg/ml, completely restored uPA protein expression levels in TGF-β1-treated cells (Fig. 4C). The transcriptional activity of uPA was also reduced following TGF-β1 treatment, while administration of APE at doses of 20 or 40 μg/ml resulted in an increase in uPA mRNA levels (Fig. 4D). These results demonstrated that APE treatment decreases PAI-1 expression and restores that of uPA.

## Discussion

Liver fibrosis is a chronic disease that is a culmination of the insidious effects of liver degeneration. In recent years, the clinical focus has concentrated predominantly on the prophylaxis and treatment of primary liver disease, including chronic hepatitis, or in trying to avoid contact with hepatotoxic substances (24–27). It has been shown that certain active components found in traditional herbal plants are effective in suppressing liver fibrosis and collagen synthesis, which indicated a potential therapeutic option for liver fibrosis (28–30). One of these studies demonstrated that APE significantly reduced levels of serum glutamic pyruvic transaminase, glutamic oxaloacetic transaminase and hydroxyproline in liver homogenates (31). Of note, APE also attenuated the pathological changes characteristic of liver fibrosis, which had been induced by CCl_4_ (16). In the present study, the results showed that APE markedly suppressed hepatic stellate cell proliferation and invasion, as well as their activation. Furthermore, these inhibitory effects may be due to the effects of APE on the TGF-β/Smad pathway.

HF results from the excessive secretion of matrix proteins by HSCs, a process which is primarily triggered by TGF-β1. A recent study suggested that garlic extract exhibits therapeutic effects in liver fibrosis through inhibition of TGF-β1 in HSCs (32). Other studies have demonstrated that proanthocyanidin from grape seed extract exerts protective hepatocellular effects, which resulted in the amelioration of murine liver fibrosis, induced by TGF-β1 (33). In addition, using TGF-β1 small interfering RNA to inhibit the expression of TGF-β1 attenuated rat hepatic fibrosis induced by a high-fat diet and CCl_4_ (34). The results of the present study confirmed that administration of TGF-β1 significantly increased collagen synthesis in HSCs, which was hypothesized to be involved in the formation of liver fibrosis. APE suppressed the proliferation induced by FBS and the cell invasion induced by TGF-β1 in HSCs in a dose-dependent manner. In addition, APE reduced the collagen synthesis and the expression of α-SAM in HSCs, also in a dose-dependent manner. These results indicated that TGF-β1 exerts pro-fibrotic activity in HSCs, primarily through a dual action of collagen synthesis promotion and the inhibition of collagen degradation. These combined effects ultimately lead to excessive collagen deposition.

TGF-β1 controls a diverse set of cellular process and its canonical signaling is mediated via the TGF-β-induced phosphorylation of receptor-activated Smad2 and Smad3 (35). It was recently shown that the expression of the phosphorylated Smad2 and Smad3 proteins was higher in samples of CCl_4_-induced liver fibrosis, compared with that in the controls (36). Furthermore, overexpression of Smad ubiquitin regulatory factor 2 suppressed TGF-β-mediated liver fibrosis (37). Finally, co-treatment with *Boswellia serrata* and *Salvia miltiorrhiza* extracts reduced dimethylnotrosamine-induced hepatic fibrosis in mice via downregulation of phosphorylated Smad3 (38). The present study showed that APE inhibited the phosphorylation of Smad2 and Smad3 which was induced by TGF-β1 in HSCs, in a dose-dependent manner. It was therefore hypothesized that the reduction of phosphorylated Smad2 and -3 by APE may underlie its inhibitory effects on HSCs.

Smad7 serves as the negative feedback regulator for TGF-β signaling, acting to antagonize the activity of the receptor-regulated Smads, which leads to a termination of the TGF-β signal (39). Suppression of Smad7 by DNA methyltransferase 1 promoted the phosphorylation of Smad2 and Smad3 that had been induced by HSC activation, or liver fibrosis in general (40). Recent studies have shown that hepatocytes are more sensitive to the effects of TGF-β. This resulted in enhanced cell death in S7DeltaE1 and wild-type mice that had a deletion of exon I from the endogenous Smad7 gene. A study demonstrated that hepatocytes are more sensitive to the effects of TGF-β. In these mice, an increase in oxidative stress and an increase in cell damage in response to CCl_4_ was observed (41). Smad7-overexpression in common bile duct ligation rats reduced the expression of collagen and α-SMA, as well as the hydroxyproline content in the liver (42). The present study also showed that TGF-β1-induced reduction in Smad7 expression was ameliorated by treatment with APE in a dose-dependent manner. Smad7 has recently been used successfully to abrogate TGF-β signaling in a number of fibrotic diseases, resulting in decreased collagen or α-SMA expression as a result of inhibition of the phosphorylation of Smads (43). Therefore, it is possible that the inhibition of Smad activation caused by the application of APE may be partly due to an upregulation in Smad7 expression, with a resultant reduction in collagen production.

uPA and plasmin are involved in the cellular proteolytic degradation of ECM proteins and in the maintenance of tissue homeostasis. The activities of uPA/plasmin predominantly rely on the functioning of a potent inhibitor of PA, PAI-1 (44). Under normal physiological conditions, PAI-1 controls the activities of the uPA/plasmin system and thus maintains tissue homeostasis. Recently, a number of studies have demonstrated that co-expression of Smad7 and uPA attenuates CCl_4_-induced liver fibrosis in rats (45,46). Furthermore, the administration of Genistein was shown to modify liver fibrosis and improve liver function by inducing uPA expression in CCl_4_-treated rats (47). PAI-1 levels are known to be significantly elevated in fibrotic tissues and a lack of PAI-1 protects certain organs from fibrosis in response to injury-associated, pro-fibrotic signals (48). Liver fibrosis produces intrinsically high levels of PAI-1 and low levels of urokinase-type plasminogen activator. This altered ratio of activator and inhibitor activities is an important factor contributing to altered fibrin degradation and the subsequent ECM metabolism. The imbalance ultimately aids in the formation of liver fibrosis (49). In the present study, TGF-β1 was shown to increase mRNA and protein levels of PAI-1 and decrease those of uPA, while APE significantly suppressed TGF-β1-induced PAI-1 expression and increased uPA expression in the presence of TGF-β1 in a dose-dependent manner, therefore reversing the effects of TGF-β1. In view of growing evidence suggesting that elevated PAI-1 expression and reduced uPA expression are essential in collagen accumulation in HSCs, the APE-induced decrease in PAI-1 mRNA and protein expression may interfere with collagen deposition, thus preventing keloid formation.

In conclusion, the present study demonstrated that APE inhibited cell proliferation, invasion and collagen synthesis in HSCs, and that the mechanisms underlying these effects may involve the TGF-β/Smad signaling pathway. These results provided evidence for the promising potential therapeutic use of APE in liver fibrosis.

## Figures and Tables

**Table I:** Effects of APE on viability of HSCs treated with 10% FBS.

Group	APE dose (μg/ml)	Absorbance (A_570_)	Inhibition (%)
Control	-	0.48±0.05	-
FBS (12.5%)	-	1.04±0.1^c^	-
APE	5	0.91±0.07^a^	18.03
	10	0.83±0.05^a^	29.46
	20	0.73±0.04^b^	43.68
	40	0.51±0.04^b^	78.42
	80	0.48±0.04^b^	90.63

a P<0.05;

b P<0.01, compared with model group; and

c P<0.01, compared with control.

Data are presented as the mean ± standard deviation. APE, *Astragalus* and *Paeoniae* Radix Rubra; HSC, hepatic stellate cell; FBS, fetal bovine serum.
